# Write, draw, show, and tell: a child-centred dual methodology to explore perceptions of out-of-school physical activity

**DOI:** 10.1186/s12889-016-3005-1

**Published:** 2016-04-14

**Authors:** Robert J. Noonan, Lynne M. Boddy, Stuart J. Fairclough, Zoe R. Knowles

**Affiliations:** The Physical Activity Exchange, Research Institute for Sport and Exercise Sciences, Liverpool John Moores University, 62 Great Crosshall Street, Liverpool, L3 2AT UK; Department of Sport and Physical Activity, Edge Hill University, Ormskirk, UK; Department of Physical Education and Sport Sciences, University of Limerick, Limerick, Ireland

**Keywords:** Physical activity, Children, Write draw show and tell, Parents, Independent mobility

## Abstract

**Background:**

Research to increase children’s physical activity and inform intervention design has, to date, largely underrepresented children’s voices. Further, research has been limited to singular qualitative methods that overlook children’s varied linguistic ability and interaction preference. The aim of this study was to use a novel combination of qualitative techniques to explore children’s current views, experiences and perceptions of out-of-school physical activity as well as offering formative opinion about future intervention design.

**Methods:**

Write, draw, show and tell (WDST) groups were conducted with 35 children aged 10–11 years from 7 primary schools. Data were analysed through a deductive and inductive process, firstly using the Youth Physical Activity Promotion Model as a thematic framework, and then inductively to enable emergent themes to be further explored. Pen profiles were constructed representing key emergent themes.

**Results:**

The WDST combination of qualitative techniques generated complimentary interconnected data which both confirmed and uncovered new insights into factors relevant to children’s out-of-school physical activity. Physical activity was most frequently associated with organised sports. Fun, enjoyment, competence, and physical activity provision were all important predictors of children’s out-of-school physical activity. Paradoxically, parents served as both significant enablers (i.e. encouragement) and barriers (i.e. restricting participation) to physical activity participation. Some of these key findings would have otherwise remained hidden when compared to more traditional singular methods based approaches.

**Conclusions:**

Parents are in a unique position to promote health promoting behaviours serving as role models, physical activity gatekeepers and choice architects. Given the strong socialising effect parents have on children’s physical activity, family-based physical activity intervention may offer a promising alternative compared to traditional school-based approaches. Parents' qualitative input is important to supplement children’s voices and inform future family-based intervention design. The WDST method developed here is an inclusive, interactive and child-centred methodology which facilitates the exploration of a wide range of topics and enhances data credibility.

## Background

Regular physical activity is associated with wide-ranging health benefits for children [1, 2] and averts the onset of a range of non-communicable diseases, including obesity, type II diabetes and cardiovascular disease [3, 4]. Despite these well-established health outcomes few UK children currently achieve the recommended levels of physical activity to benefit their health (60 daily minutes of moderate to vigorous intensity physical activity; MVPA) [5]. Physical activity promotion is therefore essential within childhood with physical activity levels in childhood influencing lifestyle choices in adulthood and active children tend to be active adults [6–8].

**Fig. 1:**
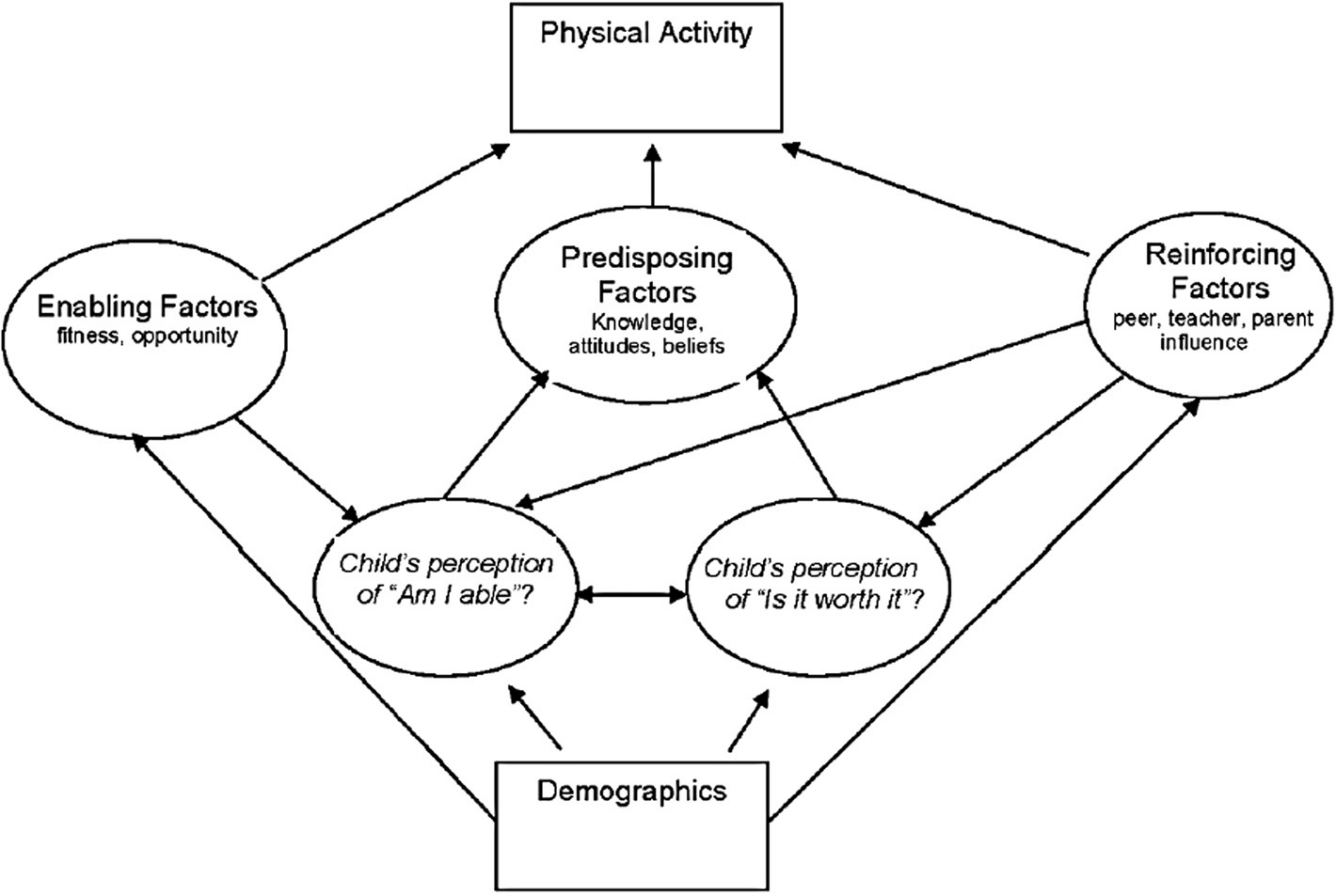
Conceptual diagram of the YPAPM (Welk, 1999)

Theoretical models are used as a framework to understand factors that enable or restrict physical activity [9]. These multiple influences are presented in the Youth Physical Activity Promotion Model (YPAPM; Fig. 1) [10], a socio-ecological model based on the fundamental principles of the PRECEDE-PROCEED model [11]. The model’s socio-ecological approach embraces an appreciation for community-level influences including the social and built environment and thus provides an appropriate conceptual framework for informing the design of intervention strategies that target changes beyond the level of the individual [9].

To date physical activity promotion strategies for young people have mainly been school-based targeting physical activity behaviours throughout the school day, yet few have reported positive health effects [12–14]. The out-of-school period (i.e., specifically after school and weekend) may offer a promising alternative during which to intervene given the precipitous decline in physical activity levels during these periods [15–17]. Moreover, the out-of-school period offers added opportunities for family based physical activity, and with parents being among the strongest influences on children’s physical activity [18, 19], serving as physical activity ‘gate keepers’, role models, and sources of support [20–22], there is potential for parents to act as catalysts to increase children’s out-of-school physical activity. Parents also govern the home environment and as such are in a key position to promote other behaviours that are conductive to children’s health [23, 24]. For example, children living in home environments that offer less support for screen time (i.e., no media equipment in children’s bedrooms and greater restricting rules), have been shown to spend significantly less time sedentary [25–27].

Ecological models postulate that health behaviours are shaped by the setting in which they occur [9, 10]. Neighbourhood environmental factors such as physical activity provision [28], proximity [29, 30], traffic volume, and neighbourhood safety [31] are considered to be important influences on children’s physical activity. Such factors are likely to influence parental perceptions of the environment and consequently the level of autonomy children are afforded by parents to be active outdoors and independent of adult supervision [32, 33]. Although time spent outdoors is consistently associated with higher daily physical activity in children [34, 35], parents often limit children’s levels of outdoor play in response to concerns about safety (i.e., road safety and ‘stranger danger’) [31, 36], even when children report positive perceptions of the local neighbourhood [37]. Restricting children to the home environment has been linked with lower levels of active transport and MVPA in Australian children [38] and more recently, greater levels of sedentary time in UK children [39].

Whilst quantitative approaches report physical activity prevalence and identify associations (e.g., [20, 32]), they provide limited contextual understanding or explanation as to why some children are more active than others, and offer little insight into intervention design. Engaging the intended user groups (i.e., children and parents) within design, eliciting their perspectives on physical activity and content is central to a phased approach to complex intervention design, and is deemed essential to their success [40, 41]. There remains however, a dearth of literature featuring the ‘children’s voice’ with qualitative research exploring children’s physical activity largely based upon data generated from parent led focus groups [42, 43] and interviews [22, 44]. Moreover, formative physical activity child intervention studies generally proceed with the informed view of what parents consider children need rather than adopting a humanistic child-led approach [45–47].

Humanism is a ‘holistic’ approach that emphasises the study of the whole child, through the eyes of the child, rather than the eyes of parents or researcher. The approach encourages children to think about their own personal feelings, and how they perceive and interpret experiences thereby offering a unique child-centred insight into the factors that drive children’s behaviour [48]. Child-led focus groups are humanistic and acknowledge children as experts [49]. They have been used before to explore children’s perspectives and attitudes towards physical activity [50, 51]. However, because children differ in cognitive and linguistic ability, interaction preference, and experience similar events in rather different ways, a more developmentally appropriate and creative methodology than focus groups may be needed [52, 53].

Participatory visual methods such as write and draw and its variations are highly efficient and ethically compliant research methods that are particularly suited for research with children for reasons of inclusivity and interactivity [54, 55]. Write and draw is popular in child-focused health research [56–58] and has been used recently to explore children’s physical activity beliefs [59] and playground experiences [60]. When compared to other qualitative approaches, drawing provides children with greater control over their expression, allowing them to reflect upon and articulate what is important to them, and the drawings themselves are rich visual illustrations that directly represent children’s perspectives and/or experiences [61, 62]. To date, research employing write and draw has somewhat focussed on drawing as representation with an emphasis on the tangible outcome, using the marks made on paper (i.e., drawing alone) or a combination of drawing and labelling as a source of data [60 58]. Such representations may not, however, be an accurate reflection of children’s intended meaning, as the interpretation of the drawing is researcher dependent and may therefore influence study credibility [63, 64].

WDST is a new method that represents an evolution of the write and draw and focus group method. The current study introduces WDST and provides a conceptual framework and practical checklist for its future application (Table 1). Contrary to that of traditional write and draw approaches, children are encouraged to articulate their own meaning embedded within their drawing and thus individual narrative commentary is formed (i.e., drawing as meaning-making) [65, 66]. Aside from providing children with greater control over their expression and recognising the social context in children’s drawing [67, 68], considering both representations together provides a more comprehensive and credible account of children’s perceptions and experiences in both an empowering and personally relevant manner [55, 69]. As a whole, the WDST method provides children with alternative ways of expression and enables a deeper exploration of children’s thoughts and perceptions by not limiting children to verbal communication. It was envisioned that the interactive and dual methods based approach (i.e., WDST) would foster greater inclusivity and would elicit more representative and detailed perceptions on out-of-school physical activity that perhaps would remain uncovered when using traditional singular methods based approaches including focus groups [65, 70–72].

**Table 1:** Write, draw, show and tell methodology process

Philosophy	• Humanistic • Children as experts • Unique perspective unnoticed from adult world
Recruitment	• Study recruitment information given • Parental consent and child assent obtained
Assent	• Verbal explanation of research purpose, processes involved and data uses • Verbal explanation of structure and context of WDST group - write/draw/storytelling etc. • Obtain verbal child assent
Setting	• Area where children can be seen but not overheard. • Circular seating arrangement with researcher sat with children. • Researcher and children address each other by first name.
Show	• Interactive ice breaker activity. • Provides children opportunity to practice speaking aloud and establishes an environment in which sharing and listening is valued. • Provide post-it note© paper and a pencil to write down responses. • Children place responses on to a flip chart board and before doing so provide a verbal account of the meaning behind written responses.
Write & Draw	• Write and draw activity. • Free access to drawing materials/no constraints on contribution or time. • Engage children in child-centred informal conversation to verify interpretation and add context to drawing. • Provide motivational comments but refrain from providing evaluation of drawings.
Tell	• Proceed with group discussion around more cognitively challenging open-ended questions. • Use terms and terminology used by children. • Ensure all children have equal opportunity to contribute. • Demonstrate genuine interest in children’s perspectives (i.e., paraphrase responses, relate responses to earlier comment or to one made by another child). • Seek clarification (i.e., probe for deeper explanations and real life examples).
Analysis	• Triangulate and pool all three data streams • Content analysis of themes • Present visual representation of drawing combined with narrative • Pen profile analysis

Research to increase physical activity in children and inform intervention design has, to date, largely underrepresented children’s voices [45–47] and been limited to singular qualitative methods that overlook children’s varied linguistic ability and interaction preference [73, 74]. An exception to this however is a recent Australian study [24] that employed a range of methods including a family interview, home tour and direct observation to explore children’s and parents’ perceptions of home physical environmental influences on children’s physical activity and sedentary time. Interviews may, however, have been prone to social desirability given that interviews were conducted in the presence of parents [75, 76]. A more detailed understanding of UK children’s perceptions of context specific physical activity, the participation barriers they face, as well as factors that support them to lead a physically active lifestyle may inform future physical activity promotion strategies including intervention design aimed at low active UK children. The aim of this study is to therefore use a combination of qualitative techniques to explore children’s current views, experiences and perceptions of out–of-school physical activity as well as offering formative opinion about future intervention design. It is envisaged that the contextual information gathered from this study will a) provide valuable insights into the meanings children ascribe towards physical activity, and b) inform the design of future out-of-school physical activity promotion strategies targeting primary school aged children.

## Methods

### Participants

Participants in this study were schoolchildren aged 10–11 years from a large north-west England city. Seven primary schools spanning a range of socio-economic areas were approached as convenience samples and agreed to participate in the study. Eligible participants had taken part in a previous cross-sectional study assessing physical activity levels [77]. Following gatekeeper consent, information packs containing child and parent information sheets and consent forms were distributed to all eligible child participants (*N* = 181) at schools to take home to parents. For the purpose of this formative study five consenting children from each school were randomly selected via lottery method to take part in a WDST group. Written informed consent and assent were obtained for 63 children (34.8 % response rate), and 35 (16 boys) of them took part in the WDST groups. Ethical approval for this study was granted by Liverpool John Moores University Ethics Committee (ref 14/SPS/033).

### Procedures

#### Write, draw, show and tell groups

WDST groups were arranged and conducted by the first author. Semi structured WDST group guides were used to ensure consistency across WDST groups, and questions were informed by the YPAPM [10]. Example WDST group questions aligned to categories of the YPAPM [10] are presented in Table 2. These questions demonstrated aspects of face validity. The research team have extensive experience working with children and conducting research on topics similar to that explored in the current study [51, 60, 78–81]. The focus group guides used in the aforementioned studies were used to inform the structure and content of the WDST guide. Prior to data collection the WDST guide was assessed independently by each of the authors after which a group meeting took place involving all authors. The WDST guide was discussed among the authors and a collective consensus was reached that the phrasing of the WDST questions and activities were age appropriate and would allow for the study aims to be achieved. One question was revised in order to improve clarity. ‘’Can you think of anything that stops you from playing outdoors” was revised to ‘’Can you think of anything that stops you from playing outdoors by your home as opposed to playing indoors?” The last author, an expert in the field as a Chartered Psychologist, provided feedback as regards age appropriateness.

**Table 2:** Example WDST Questions

YPAPM Topic	
Predisposing	Predisposing: What sorts of physical activities do you most like taking part in outside of school? Why do you like this activity more than others?
Enabling	Enabling: What sorts of things tend to stop you from doing physical activity?
Reinforcing	Reinforcing: What sorts of things do your parents or carers do that helps you be more active?

A range of qualitative techniques referred to here as WDST were incorporated into WDST groups to further stimulate children’s thinking and facilitate discussion around physical activity [82, 83] (refer to Table 1). The WDST group started with less challenging tasks and questions that children could answer as experts such as their favourite physical activities.

An ice breaker task was used at the beginning of each WDST group to provide children the opportunity to experience talking aloud to the group, and to establish an environment in which sharing and listening was valued [53]. Children were provided with post-it note© paper and a pencil and asked to write down ‘5 words to best describe physical activity to someone else’. Children subsequently placed their responses on to a flip chart board and before doing so provided a verbal explanation of the meaning behind their written responses. To allow children to express their perceptions of physical activity visually, we invited children to independently (i.e., not completed in conjunction with peers) draw an environment where they were most likely to participate in physical activity. The drawing took the focus away from direct questioning and consensus, to that of a more child-centred approach that better allowed for the lived experience to be shared [56]. Throughout the write and draw activity the first author separately engaged children in informal conversations for them to articulate what they were drawing and why.

*‘’And what about you Joe? Can you tell me what's going on in your picture?”*

With the exception of providing children with motivational comments to continue/complete as appropriate, the first author refrained from providing any evaluation of the children’s drawings. The write and draw activity provided children with greater control over their expression, allowing them to reflect upon and articulate what is important to them, and the drawings themselves are rich visual illustrations that directly represented children’s perspectives and/or experiences [61, 62]. Following the completion of the write and draw activity the WDST group proceeded with more challenging open-ended questioning around out-of-school physical activity and outdoor play. In the view that children enjoy and are satisfied most when speaking about their own personal interests and experiences the researcher provided children with various opportunities to speak about their individual physical activity interests and experiences and were encouraged to talk about themselves [84].

*Can you tell me what sorts of physical activities you most like taking part in outside of school? What is it that you like most about this activity compared to other activities? ‘Over the past week what sorts of physical activities have you done outside of school? Can you tell me where you did the activity and who it was with?*

It was anticipated that by providing children with multiple ways of expressing, ‘in their own words’, their personal perceptions and experiences it would place them at greater ease, and their increased comfort when expressing themselves would provide more honest and open discussion thereby enhancing data credibility [84, 85]. Each WDST group comprised five children and lasted 40–55 (mean = 47.7) minutes. This number of child participants has been used in previous physical activity studies undertaken by the authors and has been shown to be optimal in generating good-quality representative data [51]. Each of the WDST groups comprised children from the same school. On arrival at each school, the first author randomly selected five consenting participant names to take part in a WDST group. The names of the selected children were provided to the class teachers at schools and children were excused from class to take part. The WDST groups took place in quiet non-intrusive school class rooms where participants and researcher could be overlooked but not overheard. All WDST groups were recorded using a digital recorder and were transcribed verbatim for further analysis and anonymised. In total, 7 WDST groups were conducted resulting in 242 pages of raw transcription data, Arial font, size 12, double spaced.

#### School transport data

Active school travel contributes to children’s daily physical activity levels but is strongly influenced by household distance from school [30, 86]. To offer a more detailed insight into children’s unstructured out-of-school physical activity we assessed the prevalence of active travel as well as school-home distance. Household distance to school was objectively measured using Google maps online route planner https://www.google.co.uk/maps. The shortest route from school addresses to parent reported home addresses was used [86]. Transportation mode to school was child reported. Responses included (walk, cycle, scooter, bus, car, train, taxi, other). Responses were dichotomised into (0 reference category) active transport and (1) passive transport. Average participant travel distance from home to school was 1.51 km (Median = 0.9 km; IQR = 1.7 km).

### Data management and analysis

The WDST method generated three separate sources of data, a frequency count (show activity), visual data (write and draw activity) and verbatim data (tell activity and children’s write and draw narratives). The separate data sources were pooled together for complimentary purposes in order to expand, enhance and clarify findings from each of the separate data sources. In this case, one stage did not inform the next, rather a mixed analysis approach was taken and in doing so the analysis strands did not interact until the data interpretation stage. For the ‘show’ data, child written responses were summed to produce frequency counts.’ Tell’ data were analysed through a deductive and inductive process, firstly using the YPAPM [10] as a thematic framework reflecting the underlying study objectives, and then inductively to enable emergent themes to be further explored [87, 88]. The pen profile approach has been used in recent child physical activity research (see [51, 78] for detail) and presents findings from content analysis via a diagram of composite key emerging themes. For these reasons it is an appropriate and effective way of presenting data to researchers that have an affinity with both quantitative and qualitative approaches [60, 81].

A similar analysis approach was undertaken with the write and draw data. Drawings needed to be a legible representation of people, events, and/or places to satisfy quality standards. Children’s narratives were transcribed verbatim, classified as a written ‘report’, and subsequently appended to each individual drawing. The reports and drawings were then used in combination to categorise ‘marks’ on paper in relation to specific themes (i.e. play, games, social interaction, environment). A ‘mark’ refers to where child ‘reports’ were identifiable with a ‘theme’. In most cases each drawing identified more than one theme and thus more than one mark. For example a drawing containing a child participating in a game of football with friends would require marks for more than 1 theme (both social interaction and activity).

To ensure accuracy and allow for alternative interpretations of the data, the WDST group recordings, transcripts and drawings were independently reviewed by the second and third authors and were then cross-examined against the data in reverse, from the pen profiles to the transcripts and write and draw data sheets. This process was repeated until a 90 % agreement level had been reached by the group. Methodological rigor, credibility and transferability were achieved via verbatim transcription of data and triangular consensus procedures, and comparison of pen profiles with verbatim and illustration data accentuated dependability. In some instances visual illustrations are presented to add further context to the data. Quotations are labelled by the participant’s pseudonym, boy (B) or girl (G), and ID number. The key emergent themes identified from the data are presented first.

## Results

### Show and tell

**Fig. 2:**
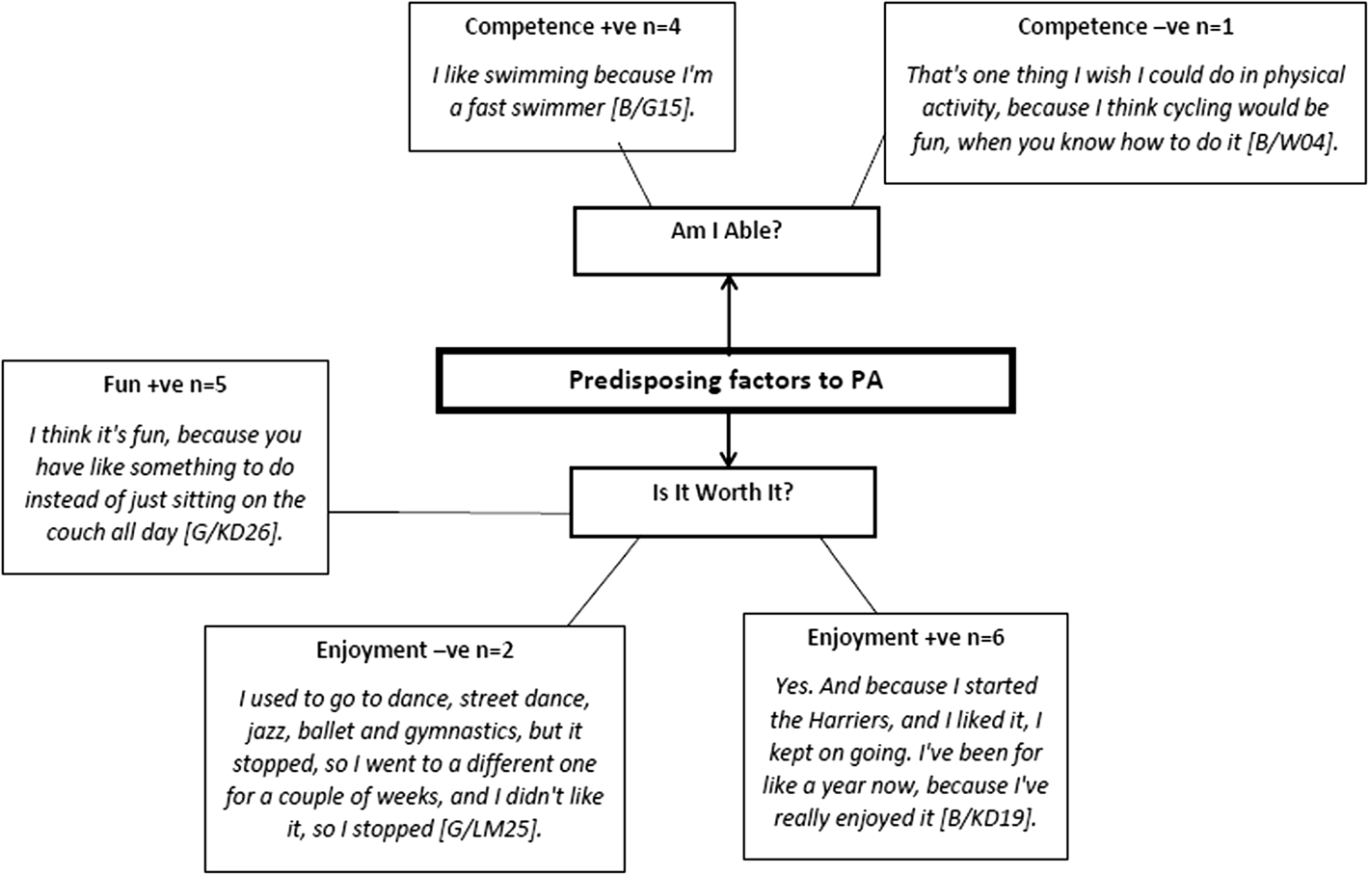
Children’s Predisposing Factors. +ve = positive. -ve = negative. M = Boy. G = Girl

In total 167 responses were reported for the show task. Physical activity was most frequently associated with organised sports (e.g., football, basketball, gymnastics) (*n* = 21), sport (*n* = 17), running (*n* = 17), swimming (*n* = 8), cycling (*n* = 3), exercise (*n* = 8), fun (*n* = 19), and health (*n* = 13). Pen profiles representing predisposing factors to physical activity are presented in Fig. 2, with two higher order themes of ‘Am I able?’ and ‘Is it worth it?’ linked to five higher order sub themes of competence + ve (*n* = 4), fun + ve (*n* = 5), enjoyment + ve (*n* = 6), competence –ve (*n* = 1), and enjoyment –ve (*n* = 2). Positive (+ve) and negative (−ve) influences featured in predisposing secondary themes.

**Fig. 3:**
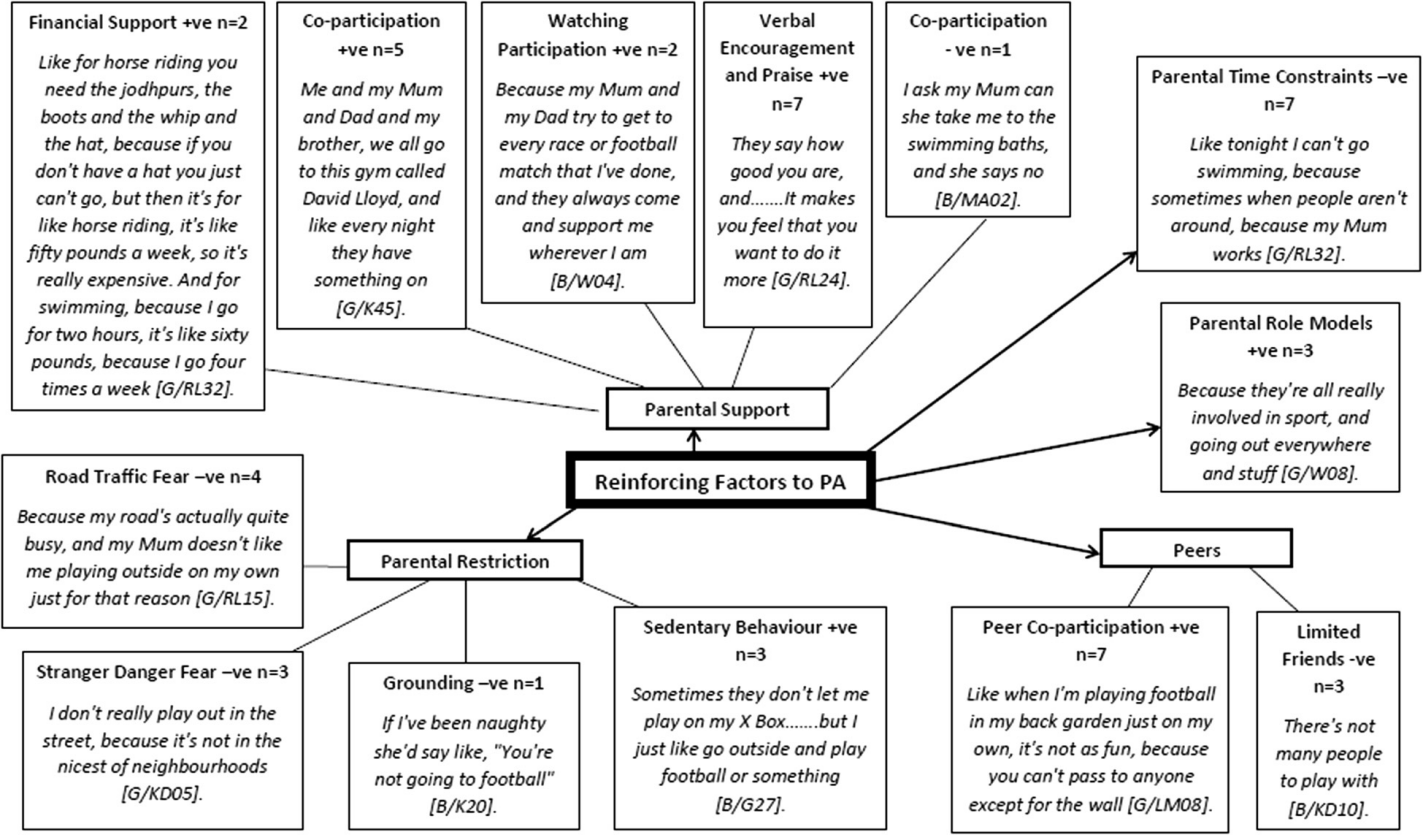
Children’s Reinforcing Factors. +ve = positive. -ve = negative. B = Boy. G = Girl

Reinforcing factors to physical activity are presented in Fig. 3, with five primary themes: parental support, parental role models, parental restriction, parental time constraints, and peers, and eleven secondary themes; financial support (*n* = 2), co-participation + ve (e.g., physical activity together) (*n* = 5), watching participation (*n* = 2), verbal encouragement and praise (*n* = 7), co-participation –ve (*n* = 1), parental role models (*n* = 3), parental time constraints (*n* = 7), peer co-participation (*n* = 7), limited friends (*n* = 3), sedentary behaviour (*n* = 3), grounding (*n* = 1), stranger danger fear (*n* = 3), and road traffic fear (*n* = 4). Positive (+ve) and negative (−ve) influences featured in both reinforcing primary and secondary themes.

**Fig. 4:**
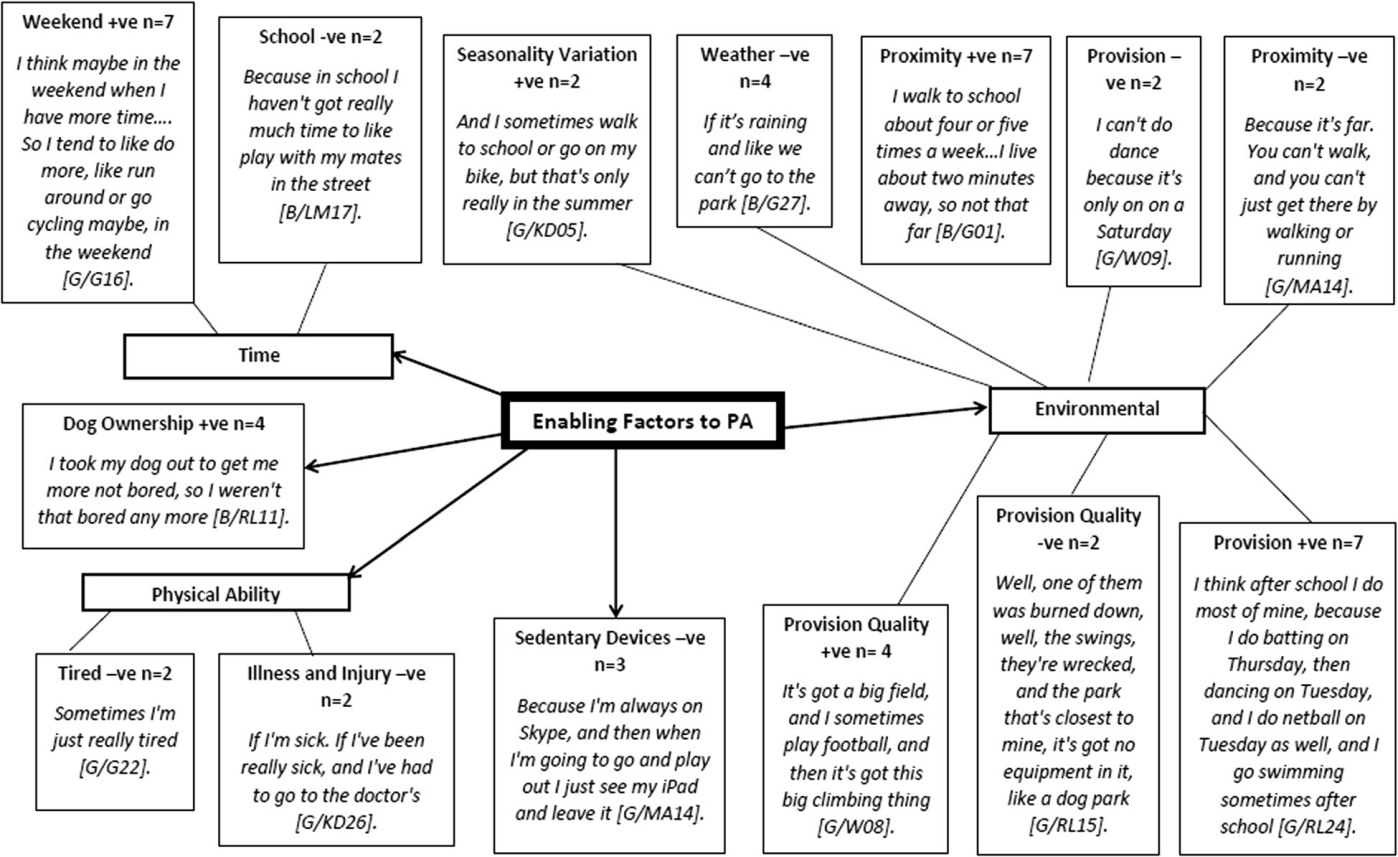
Children’s Enabling Factors. +ve = positive. -ve = negative. B = Boy. G = Girl

Enabling factors to physical activity are presented in Fig. 4. There were five primary themes; environmental factors, physical ability, time, sedentary devices and dog ownership, and twelve secondary themes: weather (*n* = 4), seasonality variation (*n* = 2), school (*n* = 2), weekend (*n* = 7), tired (*n* = 2), illness and injury (*n* = 2), proximity + ve (*n* = 7), proximity –ve (*n* = 2), provision + ve (*n* = 7), –ve (*n* = 2), provision quality + ve (*n* = 4), and provision quality –ve (*n* = 2). Positive (+ve) and negative (−ve) influences featured in enabling secondary themes.

### Write and draw

**Fig. 5:**
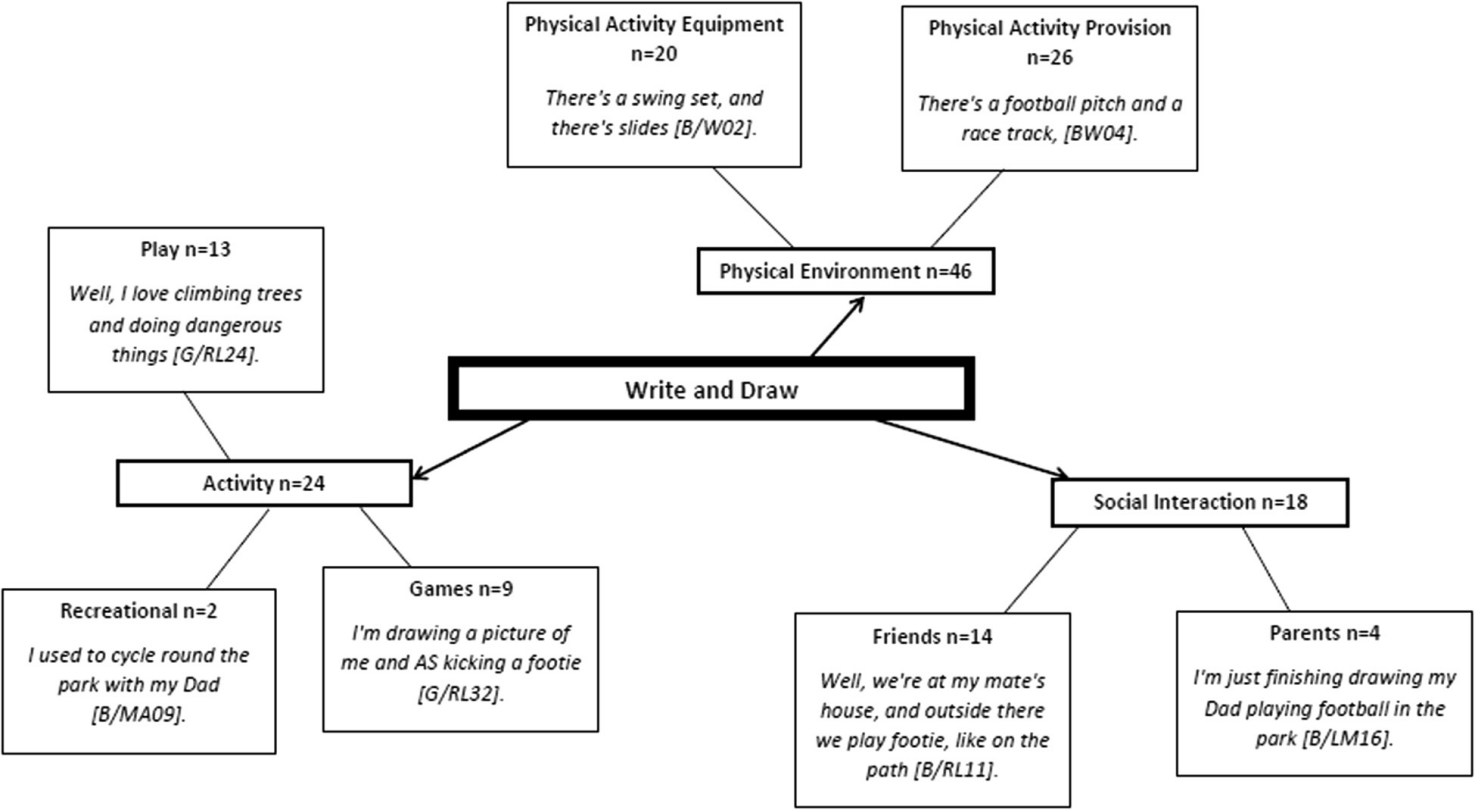
Write and Draw. +ve = positive. -ve = negative. B = Boy. G = Girl

Thirty children completed the write and draw task (14 boys), and 30 reports were extracted with 5 blank reports and 0 indefinable entries. Blank returns were due to insufficient time in one WDST group to complete the task. There were 88 marks from reports on specific themes. Figure 5 illustrates the composite pen profile with activity (*n* = 24), social interaction (*n* = 18) and physical environment (*n* = 46) as highest frequency themes. Physical activity equipment (*n* = 20), physical activity provision (*n* = 26), friends (*n* = 14), parents (*n* = 4), unstructured play (*n* = 9), games (*n* = 13), and recreational activities (*n* = 2) featured as lower order themes.

Sixty percent of children commuted actively to school. Eighty one percent and 95.2 % of these children lived within 1.0 and 2.0 km from school, respectively. The other 4.8 % lived within 3.0 km. Almost 30 % of the passive commuters lived within 1.5 km from school.

## Discussion

The primary aim of this study was to explore children’s current views, experiences and perceptions of out-of-school physical activity. Physical activity intervention design is centred on identifying factors that facilitate and inhibit children’s participation, but research featuring that of the child’s voice is presently lacking. Using children’s views, recounted experiences and perceptions of out-of-school physical activity the research presented here demonstrates how WDST may be advantageous when compared to more traditional singular methods based approaches [82]. WDST’s principal strength is its triangulation of multiple data sources which generates a rich data set representing ‘children’s voices’ and in doing so enhances data credibility strengthening the evidence on the phenomenon under investigation.

### Predisposing factors

Consistent with other studies [89, 90], children in this study principally engaged in physical activity for reasons of fun and enjoyment. Within self-determination theory (SDT) [91, 92], autonomous forms of motivation such as intrinsic motivation exist when the behaviour is viewed as enjoyable. In this study, the competitive and vigorous nature of organised physical activities appeared particularly appealing and enjoyable for many children as they perceived them to be more engaging and beneficial to physical health. In line with SDT [91, 92], children’s physical activity self-perceptions (i.e. self-efficacy and perceived competence) were both key influences on physical activity enjoyment and participation, with children expressing a sense of enjoyment towards activities that they are ‘good’ at. Children with higher self-perceptions possess higher motivation to be physically active and approach physical activity related tasks with a high expectancy of success, leading to greater perseverance and enjoyment in physical activity than children with low physical activity self-perceptions (e.g., [93, 94]). Although children’s sense of competence can be related to both perceived physical activity skill and experience, evaluative feedback from significant others, largely that of parents, but also friends, is understood to be of particular importance [95]. Alternatively, activity monitors such as pedometers provide feedback reflecting individual activity behaviour and serve as a tool to self-monitor and set personalised goals. Increasing self-efficacy by providing feedback about physical activity may effectively increase physical activity in children [96, 97].

### Enabling factors

Almost all children reported physical activity access and provision availability as key physical activity facilitators. The weekday after school period provided children with the greatest perceived access to clubs and recreational facilities and with this in mind, many children consequently determined after-school as one of their most active time periods. The weekend was also linked to high activity with greater opportunities for competitive sport participation (particularly football) and family-based activities such as bike riding and walking relative to other periods of the week. Children credited this to both them and their parents having greater discretional time to partake in physical activity on weekend days. Such findings are in contrast to recent quantitative studies that reported significant declines in physical activity during out-of-school periods compared to other periods [98, 99]. These conflicting findings could be attributed to children not accounting for the unstructured physical activity they participate in throughout the school day on the playground and their active transport to and from school.

Indeed, few children in this study accounted for engagement in unstructured forms of physical activity such as active travel, dog walking or active play, even though 60 % of participants walked to school regularly. As seen in the show data, children generally attributed physical activity with sport, which was confirmed within the write and draw data, with children expressing a greater recollection of structured physical activities, games (i.e. football) [refer to Fig. 5]. Interestingly, almost 30 % of children in this study live within 1.5 km of the school yet do not commute to school actively. Active commuting to school and to other activities is associated with improved health [100], fitness [101–103] and energy balance [104, 105], and serves as a valuable opportunity for children to significantly increase daily physical activity levels [106–108]. Increasing children’s and parents awareness of the various forms of physical activity such as active travel and unstructured play, and how these contribute to children achieving daily physical activity recommendations is warranted.

**Fig. 6:**
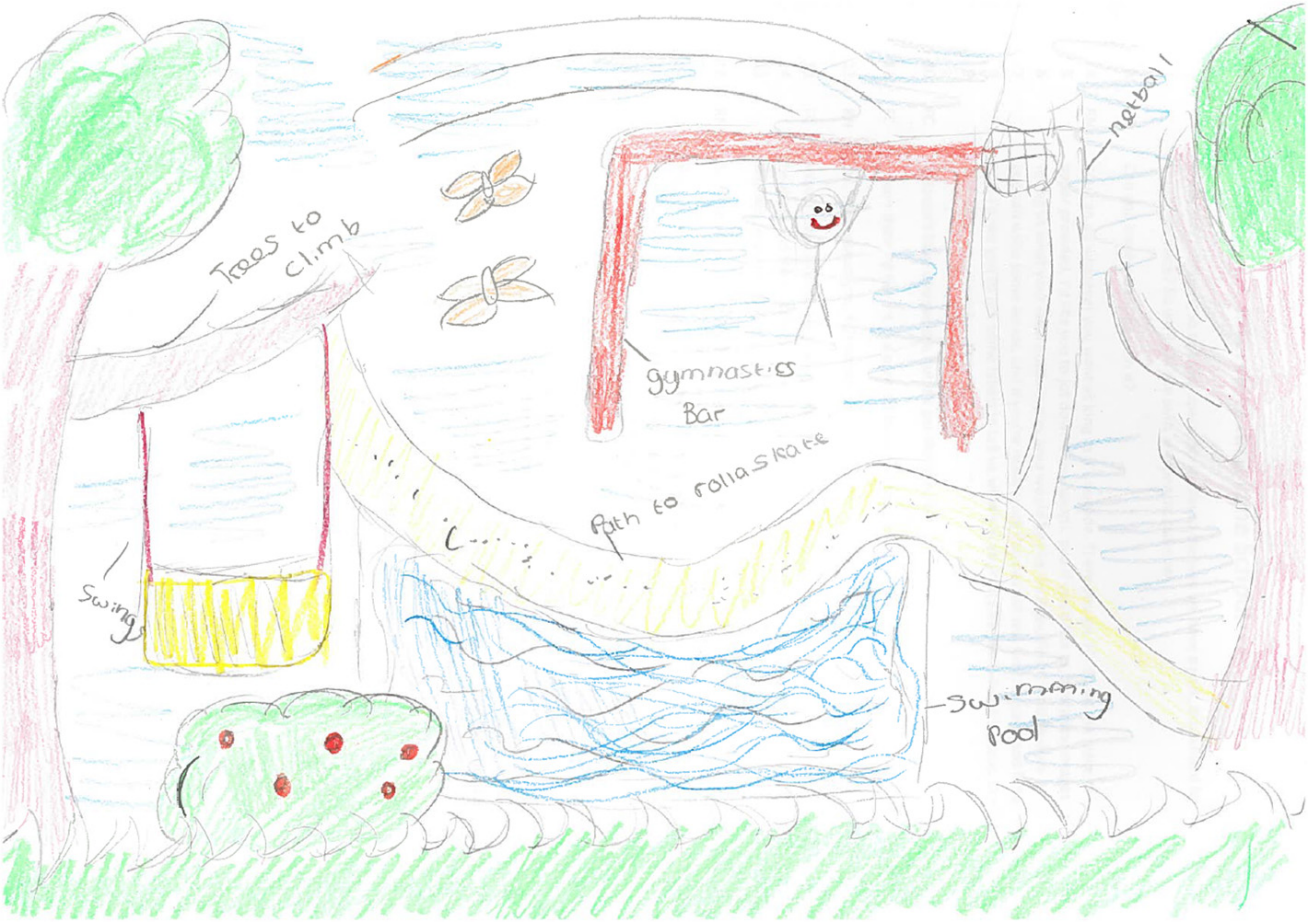
Drawing from a girl aged 11 illustrating activity equipment and provision

The visual and verbal data generated from the novel dual methodology revealed new insights and shed light on aspects of the built environment that support children’s out-of-school physical activity which may have been overlooked in previous surveys [109, 110] and singular qualitative methods based studies [73, 74]. Public parks were a popular location for physical activity but proximity to public parks influenced regular park use among children, especially unsupervised park visits. In addition to accessibility, the quality of provision and playground equipment at parks was related to children’s park use and also their experience, with children expressing a greater sense of enjoyment when there was a high prevalence of playground equipment at local public parks. Moreover, the provision of grassed areas and playground equipment appeared fundamentally important to children’s physical activity within drawings [refer to Figs. 5 and 6], so much so, many children reported travelling with parents to parks farther afield that are larger in size and have ‘better’ provision. The narrative complementing children’s drawings verified the content in drawings, and added context to the drawings by revealing insights on the reasons for the inclusion of specific content [refer to Fig. 6]. Such supplementary data would have been overlooked using traditional write and draw analysis approaches and may have influenced study findings. For example,

*I like grass, because I just think it's easy to do things on, and you can do quite a lot of things, whereas concrete, it's quite dangerous, and you could fall. And I like climbing trees…..and a gymnastics bar, because me and my sister, we use the bar where you swing, and you do like flips and stuff, because me and my sister, she's younger than me, we've got this swing, and it's high, well, about this high, and we like climbing up to the top of it and swinging, and doing flips on it, so I like that. And I'd like a netball post, because I like netball and stuff like that, and lots of bushes, so we could play hide and seek [G/RL24].*

Furthermore, although some children reported creative methods to overcome a lack of equipment, such as using clothing as goal posts, they also reported that greater availability of adequate provision (i.e. goal posts and designated green space areas) would further enhance their activity enjoyment. In order to promote regular park use among children and families a variety of features within parks may be required to support the needs of different family members [111, 112]. Future formative studies may benefit from using a similar methodology to that used here, especially those planning environmental interventions.

Proximity also influenced school transport mode in this study. Most school walkers appeared to have a high level of independent mobility, with the majority of children walking to school either alone, with siblings, or friends. While this may be because of the relatively short distance to school [30, 106], it could also be due to the presence of siblings and/or or friends which have both been associated with children’s increased independent mobility [113, 114]. In addition to the health benefits of walking to school [102], unescorted school trips could be used as a stepping stone to more broader independent mobility (i.e., outdoor play) by developing parents’ reassurances [115]. For example:

*Well, in the car you're just sitting there, and then on a bike you're actually like it's fun, and you're actually getting something from it because it's good for you, and it's better than just getting in the car and just driving [G/KD45].*

Despite home gardens/yards being a safe, popular and convenient location for children’s physical activity [110], the size of the enclosed space limited the activities that children engaged in and consequently influenced whether children used their garden/yard regularly for physical activity in this study. Although not investigated here, the absence of a garden/yard may promote greater neighbourhood play among children [116]. Consistent with previous findings, the neighbourhood environment for some children was another prime location for physical activity [117]. This was especially true for children living in cul-de-sacs and those living away from main roads, owing to higher independent mobility from parents. However, for most children unsupervised outdoor play was restricted because of parental fear regarding road traffic and children being ‘taken by strangers’. Such findings add to the existing body of evidence on social and built environmental influences [30, 33, 36, 118, 119], and in particular, cul-de-sac residency [109, 120] on children’s independent mobility and play behaviours.

Although low neighbourhood street connectivity (i.e., intersections) is associated with lower child [121] and adult walkability [122], it also reduces motorised traffic volumes, providing a safer open area for children to engage in outdoor play (e.g., football, tag) in close proximity to their home [123, 124]. Creating safe play spaces free of motorised traffic in neighbourhoods could also be an effective way of increasing children’s independent mobility and in turn increasing physical activity, partly by shaping parents’ perceptions of their children’s safety [125–127]. Such neighbourhood improvements may be particularly important for younger children and children without a garden/backyard and/or limited access to recreational green space. Moreover, providing connections between streets that are only accessible by foot rather than motorised transport may also provide a neighbourhood environment conducive to children’s play and active travel for both children and adults and should be considered by future urban planners. These findings support the need for continued traffic calming and safer route measures to alleviate parental safety concerns and support UK children’s outdoor play and active travel [128, 129].

Quantitative research has shown that children who are provided with the freedom to travel actively and play outdoors independent of adult supervision accumulate more physical activity [130, 131] and have better health than those who do not [34, 132, 133]. This study however revealed some insight into how children gain access to outdoor play and the practices used by parents to build trust and manage the perceived risks posed to children outdoors. Firstly, children in this study that were allowed to play outdoors regularly in the neighbourhood reported spatial and temporal boundaries placed on their outdoor play. For example:

*Because I can play out, but my Mum has like a thing that I have two lampposts, and I'm not allowed to go past them [B/K13].*

**Fig. 7:**
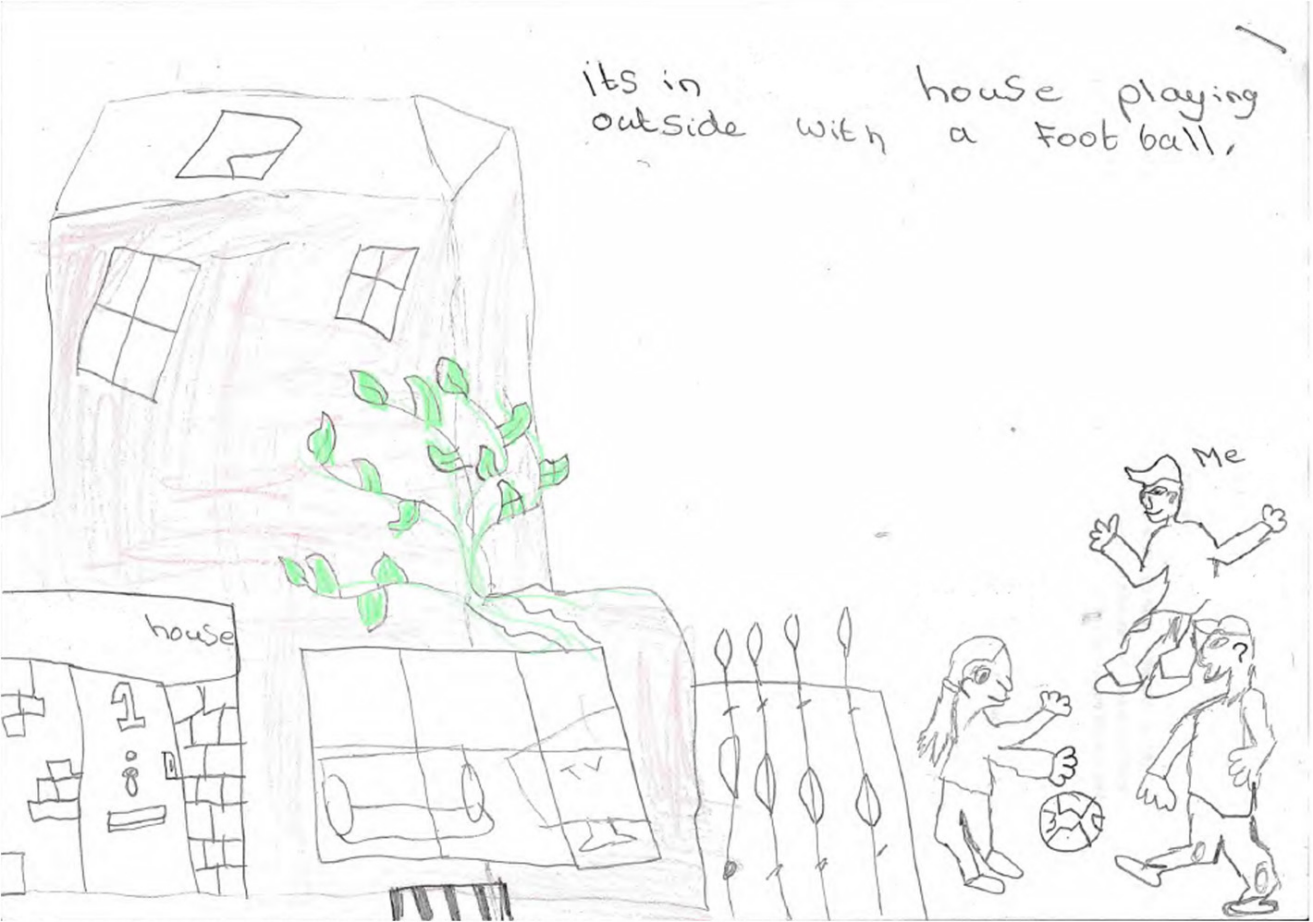
Drawing from a boy aged 10 illustrating outdoor play close to home

Moreover, children were provided with greater independent mobility when playing with friends or at nearby recreational areas [refer to Fig. 7]. The presence of other children playing out in the neighbourhood may help to reduce heightened parental neighbourhood safety concerns by way of safety in numbers [134]. Children in this study whose parents were anxious about allowing them to travel to recreational areas alone or with friends through fear of them being taken by strangers were dependent on their parents having the time and motivation to take them to recreational areas to be active. Children’s licence to play outdoors may be dependent on locally constituted beliefs about ‘good parenting’, with some parents restricting their children from playing outdoors through fear of challenging the social norm, irrespective of their own personal neighbourhood safety perceptions [135, 136]. As key gate keepers to children's outdoor play, parents' qualitative input is warranted to explore the relationship between UK children’s independent mobility and neighbourhood social norms by socio-economic background [Fig. 7 near here].

*Well, we're at my mate's house, and outside there we play footie, like on the path [B/RL11].*

### Reinforcing factors

**Fig. 8:**
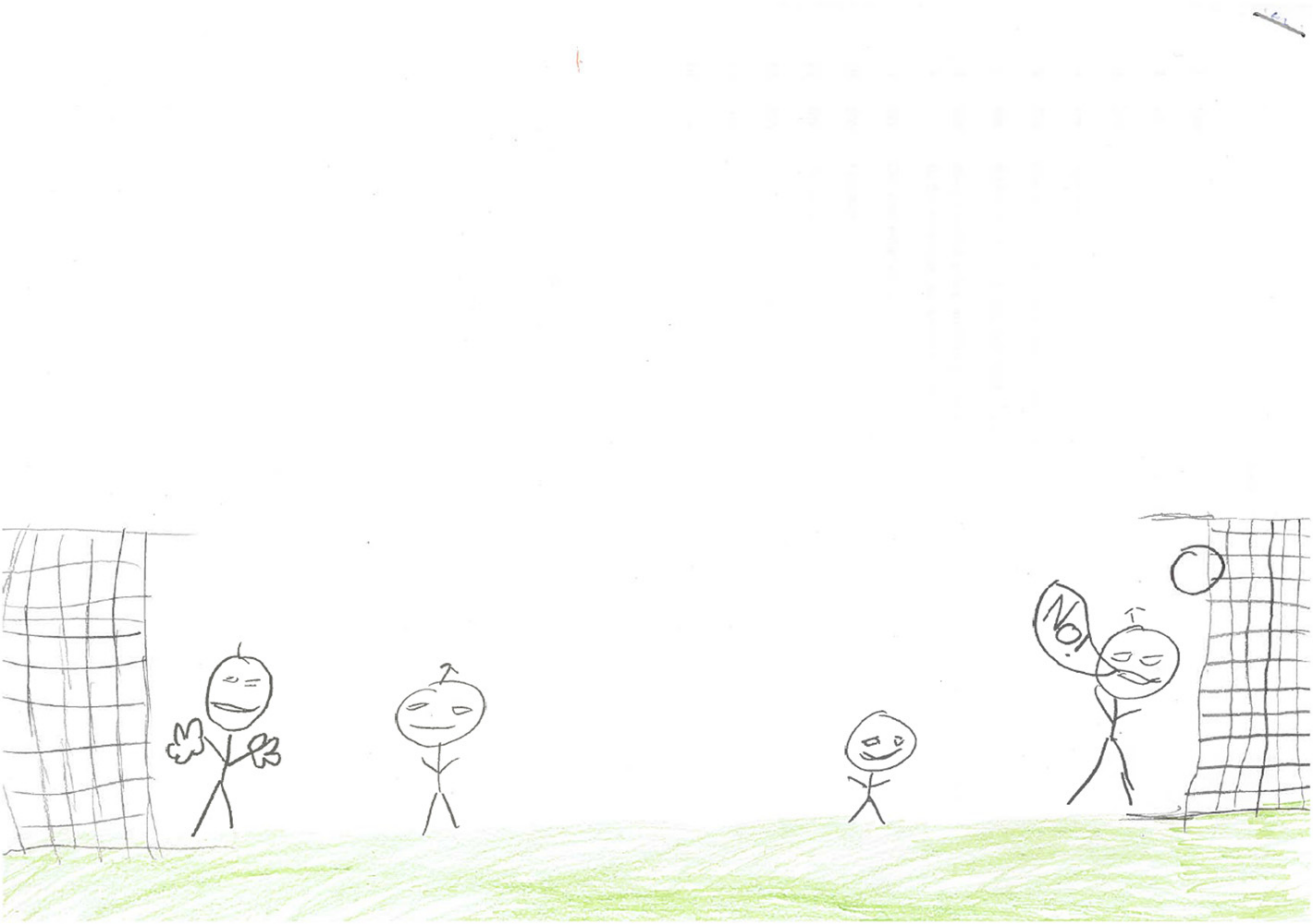
Drawing from a boy aged 11 illustrating playing football with friends

Consistent with prior quantitative research [137, 138], peer support was a key influence on children’s physical activity and the presence of friends was a central theme throughout children’s drawings [refer to Figs. 5 and 8]. The dual methods used here revealed that friends provided social support in the form of co-participation (i.e., engaging in activity together), and their presence enhanced activity enjoyment and added greater meaning to physical activity. For example, when children alluded to playing games such as football it was in the context of playing football with friends rather than playing alone.

*‘M in goal, me, and N and little D. Because they're my mates, and, like I say, I always play football with them’ [B/K20].*

Moreover, friends also played a critical role in setting children’s physical activity patterns as documented in previous quantitative studies [139, 140]. The narrative reported here however offered explanations as to why this may be. Being of similar age was important for children as it increased the likelihood of possessing similar physical activity interests. Also, outdoor play levels were dependent on other children living in the neighbourhood, with some children reporting declines in their outdoor play following friends moving home out-of-the neighbourhood, whereas others reported increased outdoor play levels following moving home to neighbourhoods where similar aged children played outdoors regularly. For example:

*‘Well, where I used to live there was loads, but because I was about six, five, and they were like nine and all that, so they didn't really want to play with me and my little sister, because we're like little, but now we've got someone called L, and she is in this class, and my sister's in Year Four, and I've got a friend who's in BS, and she's in Year Five, and then I've got RL32 and all that [G/RL15].*

Recent experimental and observational research found that the presence of friends significantly increased children’s physical activity enjoyment [141, 142], motivation [143], intensity [144], and out-of-school physical activity engagement [145]. Together, these and our findings suggest that future interventions promoting physical activity with friends and encouraging greater social interaction particularly outside of school may be a promising approach to increasing physical activity levels among UK children.

A recurring theme throughout the data was children’s significant need for parental support. Parental support is a consistent correlate of child physical activity [18, 146] but research underpinning how parental support influences children’s out-of-school physical activity is scarce. This study found that parents supported children’s physical activity in a variety of ways; however, verbal encouragement appeared to have the greatest effect on children’s emotions and their physical activity. Verbal support ranged from parents encouraging children to play outdoors instead of spending prolonged time indoors, to offering positive encouragement to children when considering ceasing physical activity participation. Both appeared to play a key role in influencing children to engage in more physical activity. Although logistical forms of support are consistent correlates of child physical activity [18, 146, 147], their limited presence within the current study data suggests that they play a less influential role on children’s physical activity relative to verbal methods. Given that parental verbal encouragement is highly amenable to change, future physical activity promotional strategies directed towards increased verbal encouragement informed by improving parental knowledge of how and where to be active in the local neighbourhood may prove useful in increasing children’s physical activity levels, particularly for children whose parents face physical, financial, or time restrictions [45, 148, 149].

It was apparent from the data that active parents, particularly fathers, were a strong motivator for children’s physical activity, despite the inconsistent relationship within the quantitative literature [150, 151]. Moreover, the direct involvement of parents in physical activities with children was also influential on children’s physical activity behaviour which supports previous findings [39, 152].

*Sunday my parents help me to do more physical activity when we go for a walk over the weekend. When I joined the Harriers (running club)…I would go with my Dad, because we both like running [B/G02].*

**Fig. 9:**
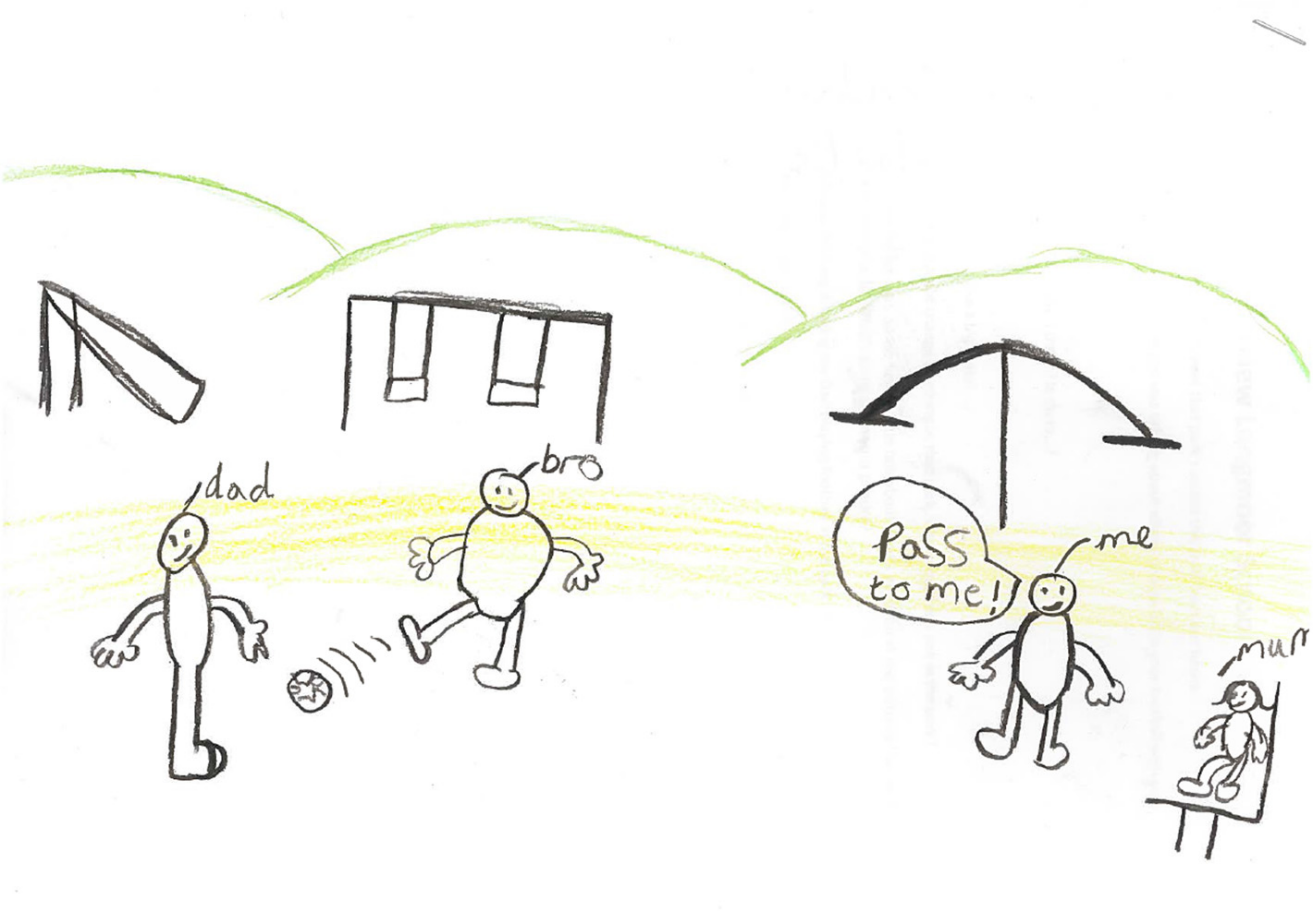
Drawing from a boy aged 10 illustrating family-based physical activity

Children’s drawings complemented such findings. Interestingly though, when parents were included in drawings it was fathers that were cited more frequently than mothers. One drawing in particular included a father engaged in physical activity with his two sons whereas mother was sitting down watching suggesting that, as portrayed here, some children may associate physical activity co-participation with fathers relative to mothers [refer to Fig. 9]. Beets and Foley [152] found that the amount of time fathers spent with their children was positively related to children’s physical activity. In light of our findings and recent qualitative [44] and experimental research [153], father-child co-participation may be an effective strategy for improving children’s physical activity. However, to appeal to all familial structures, future family-based interventions should consider encouraging parents to engage in more physical activity with their children. The weekend period may be an appropriate time to encourage physical activity between parents and children due to children not attending school and parents having fewer work responsibilities. With regards to family recreational activity, popular activities included walking, swimming and visiting public parks. Public parks play an important role in supporting physical activity, providing all families regardless of socio-economic position with the opportunity to walk, cycle, and many have specific equipment for other health enhancing physical activities [154, 155]. Promoting greater use of public parks together with information relating to fun and enjoyable activities that families can engage in together may increase park use and physical activity among families [156].

*‘I'm just finishing drawing my Dad playing football in the park. I'm going to draw some little kids playing on the park’ [B/LM16].*

Although a range of inhibiting factors including weather and school were identified by children in relation to out-of-school physical activity, the adverse influence of parents was consistent across all WDST groups. Children’s inability to access physical activity provision without the presence of their parents due to parental time constraints was a key participation barrier. Providing children and families with information on how children can best incorporate low cost physical activity into their daily lives such as walking and cycling to school or unstructured physical activity rather than structured activities that require parental presence and logistical support may be useful. Correlates research has found that children who experience fewer parental restrictions on their screen time spend significantly greater time sedentary indoors [25–27]. In this study, screen time acted as a barrier to physical activity, particularly during weekends when children had more discretionary time and autonomy over their sedentary pursuits due to no schooling and less structured activity provision. Interestingly, children reported higher levels of physical activity when parental restrictions were placed on their TV viewing and console game use in response to boredom, suggesting that parental monitoring of children’s screen-time may be another important parenting practice to target in future family-based intervention strategies.

*Sometimes they don't let me play on my X Box……., but I just like go outside and play football or something [B/G27].*

Given that parental sedentary behaviour restriction had a positive effect on children’s physical activity in this study with children opting to play outdoors, educating parents to encourage children to play outdoors more regularly with friends rather than confining children to the home environment could be a cost effective and potentially valuable means of increasing physical activity, reducing sedentary time, and improving health in UK children [157]. Advocating play and emphasising outcomes such as positive social, emotional, and cognitive well-being rather than simply the physical dangers of its absence (i.e., obesity), may resonate more strongly with parents when suggesting that their child be more active, particularly outdoors [158].

Several strengths are apparent in the present study. The development of a dual method, named here as WDST respected the expert knowledge of the children, allowed for a deeper insight of children’s experiences and perceptions, and in doing so generated a rich data set representing ‘children’s voices’ [159]. Most importantly, the combination of methods enhanced data credibility, and revealed interconnected and complementary findings on children’s views, experiences and perceptions of out-of-school physical activity that would have been overlooked via survey, adult focussed, or single qualitative methods based research. Whilst the write and draw method has been used extensively in health related research a lack of consensus around analysis has led to questions regarding its validity [54]. Alternatively, we listened to children as they drew and explored the narrative elicited from children’s drawing which recognised the social context and verified content in the drawings [65, 66]. Moreover, the triangulation of children’s drawings and supporting narrative meant that the analysis was not solely dependent upon the researcher’s interpretation of the data, and in doing so, reduced the risk of misinterpreted views, improved data credibility, and enhancing confidence in the findings [82, 160].

Further methodological strengths include the pen-profile analyses which illustrate accurately the consistency of themes in the data, rather than over-representing minority views, and the supplementary verbatim quotations verified children’s voice [161]. Moreover, the triangulation consensus of data between authors provided credibility, transferability, and dependability, and the audit trail presented here clearly outlines and justifies comprehensively the methodological decisions made throughout the study providing transparency and trustworthiness, enabling future studies to adopt a similar methodological approach [162]. In addition, this research advances previous qualitative studies by extended the literature base on children’s out-of-school physical activity by considered all components of the YPAPM [10] including the influence of peers and independent mobility, which provides new insights into an understudied area. With regards to limitations, the influence of participant bias may limit the generalisability of findings, with only 34.8 % of eligible participants providing informed consent and assent, and 19.1 % taking part.

## Conclusions

The WDST method generated complimentary interconnected data which was theoretically grounded and confirmed and uncovered new insights into factors relevant to children’s out-of-school physical activity. Specifically, the findings of this study add to our understanding of the mechanisms through which parents influence children’s activity related behaviours, and provide an insight into potential target areas for future out-of-school physical activity interventions aimed at primary school aged children. Parental involvement in future physical activity promotional strategies is essential given that paradoxically, parents served as both significant enablers (i.e. encouragement) and barriers (i.e. restricting participation) to children’s physical activity in this study. Our research concurs with that of others who report parents are physical activity gatekeepers, ‘choice architects’, and governors of the home environment and as such, are in a key position to promote behaviours that are conducive to children’s health [23, 24, 163]. Thus, parents' qualitative input is important to supplement children’s voices and inform future family-based intervention design [164]. Our findings suggest that children should be encouraged to spend more time with friends and play outdoors more. Increasing children’s levels of unstructured physical activity such as active transport and active play is warranted, but is likely to be mediated by parental license, and be dependent upon community and societal level changes to create safer neighbourhood spaces [165]. Further experimental evidence is needed to establish whether changes in parental neighbourhood perceptions positively increase children’s opportunity to engage in independent active travel and outdoor play.

We conclude that the WDST methodology developed here is an inclusive, interactive, and ethically compliant child-centred dual research method that enhances credibility by triangulating data sources and limiting researcher biases. It thus serves to benefit future researchers and practitioners aiming to elicit children’s perceptions and experiences. Further research applying WDST is needed within physical activity and health contexts to further validate its appropriateness and assist in its evolution as a child-centred method.

## Availability of data and materials

Supporting data for this study is not openly available as participants did not provide informed consent for data sharing.

## Abbreviations

B: boy
G: girl
MVPA: moderate to vigorous intensity physical activity
WDST: write, draw, show and tell
YPAPM: youth physical activity promotion model

## References

[1] Biddle SJH, Asare M (2011). Physical activity and mental health in children and adolescents: a review of reviews. Br J Sports Med.

[2] Janssen I, LeBlanc AG (2010). Systematic review of the health benefits of physical activity and fitness in school-aged children and youth. Int J Behav Nutr Phys Act..

[3] Andersen LB, Bugge A, Dencker M, Eiberg S, El–Naaman B (2011). The association between physical activity, physical fitness and development of metabolic disorders. Int J Pediatr Obes.

[4] Hills AP, Andersen LB, Byrne NM (2011). Physical activity and obesity in children. Br J Sports Med..

[5] Department of Health (2011). Start Active, Stay Active. A report on physical activity for health from the four home countries' Chief Medical Officers.

[6] Bugge A, El-Naaman B, McMurray RG, Froberg K, Andersen LB (2013). Tracking of clustered cardiovascular disease risk factors from childhood to adolescence. Pediatr Res.

[7] Marmot M (2010). Fair Society, Healthy Lives. The Marmot Review. Strategic Review of Health Inequalities in England post 2010.

[8] Telama R (2009). Tracking of Physical Activity from Childhood to Adulthood: A Review. Obes Facts..

[9] Sallis F, Owen N, Fisher EB, Glanz K, Rimer BK, Vismanath K (2008). Ecological Models of Health Behavior. Health behavior and health education: theory, research, and practice.

[10] Welk GJ (1999). The youth physical activity promotion model: A conceptual bridge theory and practice. Quest..

[11] Green LW, Kreuter MW, Deeds SG, Partidge KB (1980). Health education planning: A diagnostic approach.

[12] Kriemler S, Meyer U, Martin E, van Sluijs EMF, Andersen LB, Martin BW (2011). Effect of school-based interventions on physical activity and fitness in children and adolescents: a review of reviews and systematic update. Br J Sports Med..

[13] Metcalf B, Henley W, Wilkin T (2012). Effectiveness of intervention on physical activity of children: systematic review and meta-analysis of controlled trials with objectively measured outcomes (EarlyBird 54). BMJ..

[14] Pate RR, O’Neill JR (2009). After-school interventions to increase physical activity among youth. Br J Sports Med..

[15] Atkin AJ, Gorely T, Biddle SJH, Marshall SJ, Cameron N (2008). Critical hours: Physical activity and sedentary behavior of adolescents after school. Pediatr Exerc Sci..

[16] Atkin AJ, Gorely T, Biddle SJH, Foster C, Cavill N (2011). Interventions to promote physical activity in young people conducted in the hours immediately after school: A systematic review. Int J Behav Nutr Phys Act..

[17] Pereira S, Gomes TN, Borges A, Santos D, Souza M, dos Santos FK, Chaves RN, Katzmarzyk PT, Maia JAR (2015). Variability and Stability in Daily Moderate-to-Vigorous Physical Activity among 10 Year Old Children. Int J Environ Res Public Health..

[18] Mitchell J, Skouteris H, McCabe M, Ricciardelli LA, Milgrom J, Baur LA, Fuller-Tyszkiewicz M, Dwyer G (2012). Physical activity in young children: a systematic review of parental influences. Early Child Dev Care.

[19] Sterdt E, Liersch S, Walter U (2014). Correlates of physical activity of children and adolescents: A systematic review of reviews. Health Educ J.

[20] Crawford D, Cleland V, Timperio A, Salmon J, Andrianopoulos N, Roberts R, Giles-Corti B, Baur L, Ball K (2010). The longitudinal influence of home and neighbourhood environments on children’s body mass index and physical activity over 5 years: the CLAN study. Int J Obes..

[21] Lau EY, Barr-Anderson DJ, Dowda M, Forthofer M, Saunders RP, Pate RR (2015). Associations Between Home Environment and After-School Physical Activity and Sedentary Time Among 6th Grade Children. Pediatr Exerc Sci..

[22] O'Connor J, Brown A (2013). A qualitative study of ‘fear’ as a regulator of children's independent physical activity in the suburbs. Health Place..

[23] Maitland C, Stratton G, Foster S, Braham R, Rosenberg M (2013). A place for play? The influence of the home physical environment on children’s physical activity and sedentary behaviour. Int J Behav Nutr Phys Act.

[24] Maitland C, Stratton G, Foster S, Braham R, Rosenberg M (2014). The Dynamic Family Home: a qualitative exploration of physical environmental influences on children’s sedentary behaviour and physical activity within the home space. Int J Behav Nutr Phys Act..

[25] Brindova D, Pavelka J, Ševčikova A, Žežula I, van Dijk JP, Reijneveld SA, Geckova AM (2014). How parents can affect excessive spending of time on screen-based activities. BMC Public Health..

[26] Carlson SA, Fulton JE, Lee SM, Foley JT, Heitzler C, Huhman M (2010). Influence of Limit-Setting and Participation in Physical Activity on Youth Screen Time. Pediatrics..

[27] Cillero IH, Jago R (2011). Sociodemographic and home environment predictors of screen viewing among Spanish school children. J Public Health.

[28] Grow HM, Saelens BE, Kerr J, Durant NH, Norman GJ, Sallis JF (2008). Where Are Youth Active? Roles of Proximity, Active Transport, and Built Environment. Med Sci Sports Exerc.

[29] Chillon P, Panter J, Corder K, Jones AP, Van Sluijs EMF (2015). A longitudinal study of the distance that young people walk to school. Health Place..

[30] D’Haese S, De Meester F, De Bourdeaudhuij I, Deforche B, Cardon G (2011). Criterion distances and environmental correlates of active commuting to school in children. Int J Behav Nutr Phys Act..

[31] Carver A, Timperio A, Crawford D (2008). Playing it safe: The influence of neighbourhood safety on children’s physical activity—A review. Health Place..

[32] D'Haese S, Timperio A, Veitch J, Cardon G, Van Dyck D, Salmon J (2013). Neighborhood perceptions moderate the association between the family environment and children's objectively assessed physical activity. Health Place..

[33] De Meester F, Van Dyck D, De Bourdeaudhuij I, Cardon G (2014). Parental perceived neighborhood attributes: associations with active transport and physical activity among 10–12 year old children and the mediating role of independent mobility. BMC Public Health..

[34] Gray C, Gibbons R, Larouche R, Sandseter EBH, Bienenstock A, Brussoni M, Chabot G, Herrington S, Janssen I, Pickett W, Power M, Stanger N, Sampson M, Tremblay MS (2015). What Is the Relationship between Outdoor Time and Physical Activity, Sedentary Behaviour, and Physical Fitness in Children? A Systematic Review. Int J Environ Res Public Health..

[35] McMinn AM, Griffin SJ, Jones AP, van Sluijs EMF (2013). Family and home influences on children’s after-school and weekend physical activity. Eur J Public Health.

[36] Lee H, Tamminen KA, Clark AM, Slater L, Spence JC, Holt NL (2015). A meta-study of qualitative research examining determinants of children’s independent active free play. Int J Behav Nutr Phys Act..

[37] Timperio A, Crawford D, Telford A, Salmon J (2004). Perceptions about the local neighborhood and walking and cycling among children. Prev Med..

[38] Carver A, Timperio A, Hesketh K, Crawford D (2010). Are children and adolescents less active if parents restrict their physical activity and active transport due to perceived risk?. Soc Sci Med..

[39] Atkin AJ, Corder K, Ekelund U, Wijndaele K, Griffin SJ, van Sluijs EMF (2013). Determinants of Change in Children’s Sedentary Time. PLOS ONE..

[40] Craig P, Dieppe P, Macintyre S, Michie S, Nazareth I, Petticrew M (2008). Developing and evaluating complex interventions: the new Medical Research Council guidance. BMJ..

[41] Davison KK, Jurkowski JM, Li K, Kranz S, Lawson HA (2013). A childhood obesity intervention developed by families for families: results from a pilot study. Int J Behav Nutr Phys Act..

[42] Eyre ELJ, Duncan MJ, Birch SL, Cox VM (2014). Low socio-economic environmental determinants of children’s physical activity in Coventry, UK: A Qualitative study in parents. Prev Med Rep..

[43] Hesketh KD, Hinkley T, Campbell KJ (2012). Children’s physical activity and screen time: qualitative comparison of views of parents of infants and preschool children. Int J Behav Nutr Phys Act..

[44] Zahra J, Sebire SJ, Jago R (2015). “He’s probably more Mr. sport than me” – a qualitative exploration of mothers’ perceptions of fathers’ role in their children’s physical activity. BMC Pediatr..

[45] Bentley GF, Goodred JK, Jago R, Sebire SJ, Lucas PJ, Fox KR, Stewart-Brown S, Turner KM (2012). Parents’ views on child physical activity and their implications for physical activity parenting interventions: a qualitative study. BMC Pediatr..

[46] De Lepeleere S, DeSmet A, Verloigne M, Cardon G, De Bourdeaudhuij I (2013). What practices do parents perceive as effective or ineffective in promoting a healthy diet, physical activity, and less sitting in children: parent focus groups. BMC Public Health..

[47] Jago R, Steeds JK, Bentley GF, Sebire SJ, Lucas PJ, Fox KR, Stewart-Brown S, Turner KM (2012). Designing a physical activity parenting course: Parental views on recruitment, content and delivery. BMC Public Health..

[48] Morse JM (2012). Qualitative Health Research: Creating a New Discipline.

[49] Greene S, Hogan D, Greene S, Hogan D (2005). Researching Children's Experience: Exploring Children's Views through Focus Groups. Researching Children's Experience.

[50] Lassetter JH, Ray G, Driessnack M, Williams M (2015). Consulting with children in the development of self-efficacy and recall tools related to nutrition and physical activity. J Spec Pediatr Nurs..

[51] Mackintosh KA, Knowles ZR, Ridgers ND, Fairclough SJ (2011). Using Formative Research to Develop CHANGE!: A Curriculum-based Physical Activity Promoting Intervention. BMC Public Health..

[52] Feldman RS (2011). Development across the lifespan.

[53] Gibson JE (2012). Interviews and Focus Groups With Children: Methods That Match Children’s Developing Competencies. J Fam Theory Rev.

[54] Angell C, Alexander J, Hunt JA (2015). ‘Draw, write and tell’: A literature review and methodological development on the ‘draw and write’ research method. J Early Child Res..

[55] Literat I (2013). “A Pencil for Your Thoughts”: Participatory Drawing as a Visual Research Method with Children and Youth. Int J Qual Methods.

[56] Horstman M, Aldiss S, Richardson A, Gibson F (2008). Methodological Issues When Using the Draw and Write Technique With Children Aged 6 to 12 Years. Qual Health Res..

[57] Kostmann E, Nilsson L (2012). Children’s Perspectives on Health: What Makes Children Feel Good According to Themselves?. Int J Educ.

[58] McWhirter J (2014). The draw and write technique as a versatile tool for researching children's understanding of health and well-being. Int J Health Promot Educ.

[59] Cammisa M, Montrone R, Caroli M (2011). Development and results of a new methodology to perform focus group with preschool children on their beliefs and attitudes on physical activity. Int J Pediatr Obes.

[60] Knowles ZR, Parnell D, Stratton G, Ridgers ND (2013). Learning From the Experts: Exploring Playground Experience and Activities Using a Write and Draw Technique. J Phys Act Health..

[61] Enright E, O'Sullivan M (2012). Producing different knowledge and producing knowledge differently’: rethinking physical education research and practice through participatory visual methods. Sport Educ Soc.

[62] Gabhainn SN, Kelleher C (2002). The Sensitivity of the draw and write technique. Health Educ.

[63] Cox S (2005). Intention and meaning in young children’s drawing. JADE.

[64] Einarsdottir J, Dockett S, Perry B (2009). Making meaning: Children’s perspectives expressed through drawings. Early Child Dev Care.

[65] Dockett S, Perry B (2005). Children’s drawings: Experiences and expectations of school. IJEIEC.

[66] Angell RJ, Angell C (2013). ‘More than just ‘snap, crackle and pop’: Outlining ‘DWT’ as an innovative method for research with younger children. J Advert Res..

[67] Anning A (2002). Conversations around young children’s drawing: The impact of the beliefs of significant others at home and school. JADE.

[68] Harcourt D (2011). An encounter with children: seeking meaning and understanding about childhood. Eur Early Child Educ Res J.

[69] Tay-Lim J, Lim S (2013). Privileging Younger Children’s Voices in Research: Use of Drawings and a Co-Construction Process. Int J Qual Methods.

[70] Dockett S, Perry B (2007). Trusting children’s accounts in research. J Early Child Res.

[71] Gibson F (2007). Conducting focus groups with children and young people: strategies for success. J Res Nurs..

[72] Morgan M, Gibbs S, Maxwell K, Britten N (2007). Hearing children’s voices: methodological issues in conducting focus groups with children aged 7–11 years. Qual Res..

[73] Brockman R, Jago R, Fox KR, Thompson JL, Cartwright K, Page AS (2009). "Get off the sofa and go and play": Family and socioeconomic influences on the physical activity of 10–11 year old children. BMC Public Health..

[74] Stanley RM, Boshoff K, Dollman J (2012). A Qualitative Exploration of the “Critical Window”: Factors Affecting Australian Children’s After-School Physical Activity. J Phys Act Health..

[75] Havermans N, Vanassche S, Matthijs K. Methodological Challenges of Including Children in Family Research: Measurement Equivalence, Selection Bias and Social Desirability. Child Indic Res. 2014; doi:10.1007/s12187-014-9275-1

[76] Krumpal I (2013). Determinants of social desirability bias in sensitive surveys: A literature review. Qual Quant.

[77] Noonan RJ, Boddy LM, Knowles ZR, Fairclough SJ (2016). Cross-sectional associations between high-deprivation home and neighbourhood environments, and health-related variables among Liverpool children. BMJ Open.

[78] Boddy LM, Knowles ZR, Davies IG, Warburton GL, Mackintosh KA, Houghton L, Fairclough SJ (2012). Using Formative Research to Develop the Healthy Eating Component of the CHANGE! School-based Curriculum Intervention. BMC Public Health.

[79] Downs SJ, Boddy LM, Knowles ZR, Fairclough SJ, Stratton G (2013). Exploring opportunities available and perceived barriers to physical activity engagement in children and young people with Down syndrome. Eur J Spec Needs Educ.

[80] Porcellato L, Knowles Z, Knowles Z, Gilborne D, Cropley B, Dugdill L (2014). Reflecting forward: exploring reflective methodologies with and for children. In Reflective Practice. The Sport and Exercise Sciences: Contemporary Issues.

[81] Ridgers ND, Knowles Z, Sayers J (2012). Play in the natural environment: a child focused evaluation of forest school. Child Geogr..

[82] Darbyshire P, MacDougall C, Schiller W (2005). Multiple methods in qualitative research with children: more insight or just more?. Qual Res.

[83] Pearce A, Kirk C, Cummins S, Collins M, Elliman D, Connolly AM, Law C (2009). Gaining children’s perspectives: A multiple method approach to explore environmental influences on healthy eating and physical activity. Health Place..

[84] Carnegie D (2006). How to Win Friends and Influence People.

[85] Glenn NM, Knight CJ, Holt NL, Spence JC (2013). Meanings of play among children. Childhood.

[86] Van Dyck D, De Bourdeaudhuij I, Cardon G, Deforche B (2010). Criterion distances and correlates of active transportation to school in Belgian older adolescents. Int J Behav Nutr Phys Act..

[87] Biddle SJH, Markland D, Gilbourne D, Chatzisarantis NLD, Sparkes AC (2001). Research methods in sport and exercise psychology: quantitative and qualitative issues. J Sports Sci.

[88] Smith B, Caddick N (2012). Qualitative methods in sport: a concise overview for guiding social scientific sport research. Asia Pac J Sport Soc Sci.

[89] Sebire SJ, Jago R, Fox KR, Edwards MJ, Thompson JL (2013). Testing a self-determination theory model of children’s physical activity motivation: a cross-sectional study. Int J Behav Nutr Phys Act..

[90] Timperio AF, van Stralen MM, Brug J, Bere E, Chinapaw MJM, De Bourdeaudhuij I, Jan N, Maes L, Manios Y, Moreno LA, Salmon J, Te Velde SJ (2013). Direct and indirect associations between the family physical activity environment and sports participation among 10–12 year-old European children: testing the EnRG framework in the ENERGY project. Int J Behav Nutr Phys Act..

[91] Deci EL, Ryan RM (1985). Intrinsic Motivation and Self-Determination in Human Behaviour.

[92] Deci EL, Ryan RM (2000). The “What” and “Why” of Goal Pursuits: Human Needs and the Self-Determination of Behavior. Psychol Inq.

[93] Craggs C, van Sluijs EMF, Corder K, Panter JR, Jones AP, Griffin SJ (2011). Do children’s individual correlates of physical activity differ by home setting?. Health Place..

[94] Van der Horst K, Paw MJ, Chin A, Twisk JWR, Van Mechelen W (2007). A brief review on correlates of physical activity and sedentariness in youth. Med Sci Sport Exer..

[95] Ryan RM, Williams GC, Patrick H, Deci EL (2009). Self-Determination Theory and Physical Activity: The Dynamics of Motivation in Development and Wellness. Hell J Psychol.

[96] Horne PJ, Hardman CA, Lowe CF, Rowlands AV (2009). Increasing children's physical activity: a peer modelling, rewards and pedometer-based intervention. Eur J Clin Nutr..

[97] Lubans DR, Morgan PJ, Tudor-Locke C (2009). A systematic review of studies using pedometers to promote physical activity among youth. Prev Med..

[98] Telford RM, Telford RD, Cunningham RB, Cochrane T, Davey R, Waddington G (2013). Longitudinal patterns of physical activity in children aged 8 to 12 years: the LOOK study. Int J Behav Nutr Phys Act..

[99] Vander Ploeg KA, Wu B, McGavock J, Veugelers PJ (2012). Physical Activity Among Canadian Children on School Days and Nonschool Days. J Phys Act Health..

[100] Saunders LE, Green JM, Petticrew MP, Steinbach R, Roberts H (2013). What Are the Health Benefits of Active Travel? A Systematic Review of Trials and Cohort Studies. PLoS ONE.

[101] Larouche R, Saunders TJ, Faulkner GEJ, Colley R, Tremblay M (2014). Associations Between Active School Transport and Physical Activity, Body Composition, and Cardiovascular Fitness: A Systematic Review of 68 Studies. J Phys Act Health..

[102] Lubans DR, Boreham CA, Kelly P, Foster CE (2011). The relationship between active travel to school and health-related fitness in children and adolescents: a systematic review. Int J Behav Nutr Phys Act.

[103] Voss C, Sandercock G (2010). Aerobic Fitness and Mode of Travel to School in English Schoolchildren. Med Sci Sports Exerc.

[104] Mendoza JA, Watson K, Nguyen N, Cerin E, Baranowski T, Nicklas TA (2011). Active Commuting to School and Association With Physical Activity and Adiposity Among US Youth. J Phys Act Health.

[105] Mendoza JA, Liu Y (2014). Active Commuting to Elementary School and Adiposity: An Observational Study. Child Obes.

[106] Faulkner G, Stone M, Buliung R, Wong B, Mitra R (2013). School travel and children’s physical activity: a cross-sectional study examining the influence of distance. BMC Public Health..

[107] Lee C, Li L (2014). Demographic, Physical Activity, and Route Characteristics Related to School Transportation: An Exploratory Study. Am J Health Promot.

[108] Roth MA, Millett CJ, Mindell JS (2012). The contribution of active travel (walking and cycling) in children to overall physical activity levels: a national cross sectional study. Prev Med..

[109] Veitch J, Salmon J, Ball K (2008). Children’s active free play in local neighborhoods: a behavioral mapping study. Health Educ Res.

[110] Veitch J, Salmon J, Ball K (2010). Individual, social and physical environmental correlates of children’s active free-play: a cross sectional study. Int J Behav Nutr Phys Act..

[111] Edwards N, Hooper P, Knuiman M, Foster S, Giles-Corti B (2015). Associations between park features and adolescent park use for physical activity. Int J Behav Nutr Phys Act..

[112] Kaczynski AI, Potwarka LR, Saelens BE (2008). Association of Park Size, Distance, and Features With Physical Activity in Neighborhood Parks. Am J Public Health..

[113] Mackett R, Brown B, Gong Y, Kitazawa K, Paskins J (2007). Children’s Independent Movement in the Local Environment. Built Environ.

[114] Zwerts E, Allaert G, Janssens D, Wets G, Witlox F (2010). How children view their travel behaviour: a case study from Flanders (Belgium). J Transp Geogr..

[115] Stanley RM, Maher C, Dollman J (2015). Modelling the contribution of walking between home and school to daily physical activity in primary age children. BMC Public Health..

[116] Aarts MJ, Wendel-Vos W, van Oers HAM, van de Goor LAM, Schuit AJ (2010). Environmental Determinants of Outdoor Play in Children A Large-Scale Cross-Sectional Study. Am J Prev Med.

[117] Barron C (2013). Physical activity play in local housing estates and child wellness in Ireland. Int J Play.

[118] Janssen I (2014). Crime and perceptions of safety in the home neighborhood are independently associated with physical activity among 11–15 year olds. Prev Med..

[119] Rader NE, Byrd SH, Fountain BJ, Bounds CW, Gray V, Frugé AD. We Never See Children in the Parks: A Qualitative Examination of the Role of Safety Concerns on Physical Activity among Children. J Phys Act Health. 2014; doi:http://dx.doi.org/10.1123/jpah.2014-0053 10.1123/jpah.2014-0053 25156307

[120] Hochschild TR (2012). Cul-de-sac kids. Childhood.

[121] Giles-Corti B, Wood G, Pikora T, Learnihan V, Bulsara M, Van Niel K, Timperio A, McCormack G, Villanueva K. School site and the potential to walk to school: The impact of street connectivity and traffic exposure in school neighborhoods. Health Place. 2011;17:545–50. 10.1016/j.healthplace.2010.12.011 21237697

[122] Koohsari MJ, Sugiyama T, Lamb KE, Villanueva K, Owen N (2014). Street connectivity and walking for transport: Role of neighborhood destinations. Prev Med..

[123] Mecredy G, Pickett W, Janssen I (2011). Street connectivity is negatively associated with physical activity in Canadian youth. Int J Environ Res Public Health..

[124] Tappe KA, Glanz K, Sallis JF, Zhou C, Saelens BE (2013). Children’s physical activity and parents’ perception of the neighbourhood environment: neighbourhood impact on kids study. Int J Behav Nutr Phys Act..

[125] D’Haese S, Van Dyck D, De Bourdeaudhuij I, Deforche B, Cardon G (2015). Organizing “Play Streets” during school vacations can increase physical activity and decrease sedentary time in children. Int J Behav Nutr Phys Act..

[126] Farley TA, Meriwether RA, Baker ET, Watkins LT, Johnson CC, Webber LS (2007). Safe play spaces to promote physical activity in inner-city children: results from a pilot study of an environmental intervention. Am J Health Promot..

[127] Villanueva K, Giles-Corti B, Bulsara M, Timperio A, McCormack G, Beesley B, Trapp G, Middleton N. Where Do Children Travel to and What Local Opportunities Are Available? The Relationship Between Neighborhood Destinations and Children’s Independent Mobility. Environ Behav. 2013;45(6):679–705.

[128] Randolph MJ, Benjamin RK (2014). Activating Places for Physical Activity: When “Honey Go Outside and Play” Isn’t Enough. Am J Health Promot.

[129] Stewart O, Moudon AV, Claybrooke C (2014). Multistate Evaluation of Safe Routes to School Programs. Am J Health Promot.

[130] Faulkner G, Mitra R, Buliung R, Fusco C, Stone M (2015). Children's outdoor playtime, physical activity, and parental perceptions of the neighbourhood environment. Int J Play.

[131] Schoeppe S, Duncan MJ, Badland HM, Oliver M, Browne M (2014). Associations between children’s independent mobility and physical activity. BMC Public Health..

[132] Schoeppe S, Duncan MJ, Badland H, Oliver M, Curtis C (2013). Associations of children's independent mobility and active travel with physical activity, sedentary behaviour and weight status: a systematic review. J Sci Med Sport.

[133] Stone MR, Faulkner GEJ (2014). Outdoor play in children: Associations with objectively-measured physical activity, sedentary behavior and weight status. Prev Med..

[134] Holt NL, Lee H, Millar CA, Spence JC (2015). ‘Eyes on where children play’: a retrospective study of active free play. Child Geogr..

[135] Christian HE, Klinker CD, Villanueva K, Knuiman MW, Foster SA, Zubrick SR, Divitini M, Wood L, Giles-Corti B. The Effect of the Social and Physical Environment on Children’s Independent Mobility to Neighborhood Destinations. J Phys Act Health. 2015;12 Suppl 1:S84–93. 10.1123/jpah.2014-0271 25599389

[136] Sutton L (2008). The State of Play: Disadvantage, Play and Children’s Well-Being. Soc Policy Soc.

[137] Jago R, Brockman R, Fox KR, Cartwright K, Page AS, Thompson JL (2009). Friendship groups and physical activity: qualitative findings on how physical activity is initiated and maintained among 10–11 year old children. Int J Behav Nutr Phys Act..

[138] Jago R, Macdonald-Wallis K, Thompson JL, Page AS, Brockman R, Fox KR (2011). Better with a Buddy: Influence of Best Friends on Children’s Physical Activity. Med Sci Sports Exerc.

[139] Salvy SJ, Wojslawowicz Bowker J, Roemmich JN, Romero N, Kieffer E, Paluch R, Epstein LH. Peer Influence on Children’s Physical Activity: An Experience Sampling Study. J Pediatr Psychol. 2008;33(1):39–49. 10.1093/jpepsy/jsm039 PMC2706580 17525088

[140] Gesell SB, Tesdahl E, Ruchman E. The Distribution of Physical Activity in an After-school Friendship Network. Pediatr. 2012; doi:10.1542/peds.2011-2567 10.1542/peds.2011-2567 PMC3362907 22641755

[141] Jago R, Page AS, Cooper AR (2012). Friends and Physical Activity during the Transition from Primary to Secondary School. Med Sci Sports Exerc.

[142] Salvy SJ, de la Haye K, Bowker JC, Hermans RCJ (2012). Influence of peers and friends on children's and adolescents' eating and activity behaviors. Physiol Behav..

[143] Salvy SJ, Roemmich JN, Bowker JC, Romero ND, Stadler PJ, Epstein LH (2009). Effect of Peers and Friends on Youth Physical Activity and Motivation to be Physically Active. J Pediatr Psychol.

[144] Barkley JE, Salvy SJ, Sanders GJ, Dey S, Von Carlowitz KP, Williamson ML (2014). Peer Influence and Physical Activity Behavior in Young Children: An Experimental Study. J Phys Act Health..

[145] Pearce M, Page AS, Griffin TP, Cooper AR (2014). Who children spend time with after school: associations with objectively recorded indoor and outdoor physical activity. Int J Behav Nutr Phys Act..

[146] Beets MW, Cardinal BJ, Alderman BL (2010). Parental social support and the physical activity-related behaviors of youth: a review. Health Educ Behav..

[147] Määttä S, Ray C, Roos E (2014). Associations of parental influence and 10–11-year-old children’s physical activity: Are they mediated by children’s perceived competence and attraction to physical activity?. Scand J Public Health..

[148] Edwardson CL, Gorely T (2010). Parental Influences on Different Types and Intensities of Physical Activity in Youth: A Systematic Review. Psychol Sport Exerc..

[149] Tate EB, Shah A, Jones M, Pentz MA, Liao Y, Fridlund G. Toward a Better Understanding of the Link between Parent and Child Physical Activity Levels: The Moderating Role of Parental Encouragement. J Phys Act Health. 2014; doi:http://dx.doi.org/10.1123/jpah.2014-0126. 10.1123/jpah.2014-0126 PMC5504529 25494399

[150] Biddle SJH, Atkin AJ, Cavill N, Foster C (2011). Correlates of physical activity in youth: a review of quantitative systematic reviews. Int Rev Sport Exerc Psychol..

[151] Jago R, Sebire SJ, Wood L, Pool L, Zahra J, Thompson JL, Lawlor DA. Associations between objectively assessed child and parental physical activity: a cross-sectional study of families with 5–6 year old children. BMC Public Health. 2014;14:655. 10.1186/1471-2458-14-655 PMC4091740 24970045

[152] Beets MW, Foley JT (2008). Association of father involvement and neighbourhood quality with kindergarteners’ physical activity: A multilevel structural equation model. Am J Health Promot..

[153] Morgan PJ, Collins CE, Plotnikoff RC, Callister R, Burrows T, Fletcher R, Okely AD, Young MD, Miller A, Lloyd AB, Cook AT, Cruickshank J, Saunders KL, Lubans DR. The ‘Healthy Dads, Healthy Kids’ community randomized controlled trial: A community-based healthy lifestyle program for fathers and their children. Prev Med. 2014;61:90–9. 10.1016/j.ypmed.2013.12.019 24380796

[154] Cohen DA, McKenzie TL, Sehgal A, Williamson S, Golinelli D, Lurie N (2007). Contribution of Public Parks to Physical Activity. Am J Public Health.

[155] Han B, Cohen D, McKenzie TL (2013). Quantifying the contribution of neighborhood parks to physical activity. Prev Med..

[156] Buchner DM, Gobster PH (2007). Promoting Active Visits to Parks: Models and Strategies for Transdisciplinary Collaboration. J Phys Act Health.

[157] Vandewater EA, Park SE, Hébert ET, Cummings HM (2015). Time with friends and physical activity as mechanisms linking obesity and television viewing among youth. Int J Behav Nutr Phys Act.

[158] Burdette HL, Whitaker RC (2005). Resurrecting Free Play in Young Children Looking Beyond Fitness and Fatness to Attention, Affiliation, and Affect. Arch Pediatr Adolesc Med..

[159] Kesby M (2007). Methodological insights on and from Children’s Geographies. Child Geogr..

[160] Smith J, Noble H. Bias in research. Evid Based Nurs. 2014; doi:10.1136/eb-2014-101946 10.1136/eb-2014-101946 25097234

[161] Anderson C (2010). Presenting and Evaluating Qualitative Research. Am J Pharm Educ..

[162] Carcary M (2009). The Research Audit Trial – Enhancing Trustworthiness in Qualitative Inquiry. Electron J Bus Res Methods.

[163] Thaler RH, Sunstein CR (2008). Nudge. Improving decisions about health, wealth, and happiness.

[164] Teedon P, Gillespie M, Lindsay K, Baker K (2014). Parental perceptions of the impacts the built environment has on young children's health: A qualitative examination and lay assessment amongst residents in four Scottish communities. Health Place..

[165] Little H (2015). Mothers’ beliefs about risk and risk-taking in children’s outdoor play. J Adventure Educ Outdoor Learn.

